# Notes and News

**Published:** 1888-03-10

**Authors:** 


### Notes and News.

Collected and Communicated

The next meeting of the Hospitals Association will be held at the board-room of St. Mary's Hospital, Paddington, on Wednesday, March 14th, at eight p.m., to hear and discuss a paper by Mr. Thos. Ryan, Secretary of St. Mary's Hospital (late of Queen Charlotte's), on "The Origin, History, Work, and Present State of Metropolitan Lying-in Hospitals." The chair will be taken by W. S. Playfair, Esq., M.D., LL.D. Cards of admission can be obtained on application from Mr. Howard J. Collins, Secretary, The Hospitals Association, Norfolk House, Norfolk Street, W.C.

At a meeting of the delegates of the Hospital Saturday Fund on Saturday, at the Board-room, 41, Fleet-street, the secretary (Mr. R. Frewer) announced that Mr. Augustus Harris had placed the Drury Lane Theatre at the disposal of the Hospital Saturday Convalescent Home Fund for a ticket benefit to extend from March 12th to 23rd.

The Committee of the Islington Jubilee Fund have paid over a further sum of £2,000 (making £4,000 altogether) to the new building fund of the Great Northern Central Hospital.

A bewildered correspondent of the *Oxford Chronicle*, calling himself "Pater," writes in the first place to suggest that women doctors should be employed, wherever it is practicable, for female patients. But in connexion with this he wants to know how it is that while we have women for nurses in our hospitals and homes, and women to perform the last sad offices for the dead, people cry out at the "unsexing of women" (whatever that may mean) by the study of medicine. We are quite as much at loss how to explain this and other inconsistencies of human nature as "Pater" himself can be; but we think the irrational protest against medical women is dying, if not already dead. As for his other suggestion, we feel sure that when a female gynæcologist of distinction appears she will not lack patients.

The annual meeting of governors of the City of London Hospital for Diseases of the Chest, Victoria Park, E., was held at the office, 24, Finsbury Circus, on the 21st ult., Stephen Olding, Esq., treasurer of the institution, being in the chair. The report showed that during the year 1,073 patients had been treated in the hospital, while the out-patients had numbered 15,775. The total receipts from all sources amounted to £8,089 7*s.* 3*d.*, and the expenditure to £9,810 3*s.* 3*d.*, leaving a deficit of £1,720 16*s.* The deficiency is attributable to the falling-off in the receipts from legacies, a source of income naturally very uncertain. Dr. Angel Money having resigned his post as one of the assistant physicians to the hospital, Dr. T. Glover Lyon was appointed in his place. Though the majority of the patients treated at this hospital belong to the East-end of London, there have been received there during the past year many from the provinces, besides two from Wales and two from Scotland.

It is a rule at the Samaritan Hospital for Women, Glasgow, that no one shall be present at an operation who has recently been attending an infectious case, or has come from a *post mortem* examination, or the dissecting-room. The good results of this stringent law are shown in the fact that of the 93 operations performed in the hospital during last year—almost all requiring anæsthetics, and many of them very serious in character—not one has resulted in death.

A suggestion was made at the Court of Governors of the Royal Hants County Hospitals that any subscriber residing beyond six miles from the city of Winchester should receive a letter of recommendation for one in-patient instead of the six out-patient letters that have hitherto been given them. As, however, the cost of one in-patient averages more than double the cost of six out-patients, it is difficult to see how this can be done unless the finances of the hospital are considerably increased.

At the annual meeting of subscribers to the Knackers knowle Convalescent Home, held at St. Andrew's Hall, Plymouth, the hon. secretary, Mr. R. Reynolds Fox, said that the benevolent public had as yet only partially realised the importance of convalescent homes, and this was true also of hospital managers, and those connected with working men's sick and similar societies. As a simple matter of economy, cases which would otherwise be kept an indefinitely long time on the sick list, would be accelerated to a surprising extent by removal to a convalescent home.

As the Carlisle Dispensary is suffering from lack of funds, and its governors are compelled to sell out upwards of £150 consols in order to reduce the institution's debt to the bank, the Venerable Archdeacon Prescott, who presided at the last annual meeting, suggested that, to encourage small subscriptions from the working classes, a ticket giving the right to free attendance at the dispensary should be given to every subscriber of eighteenpence. The more necessitous poor would, he contended, still be provided for by receiving tickets from wealthier subscribers, who were to receive them at the same rate—fifteen for a guinea subscription. This scheme sounds well for the dispensary, but is it fair all round? Doctors in general practice, as well as many hospital authorities, are already complaining of the number of people able to pay for medical advice who take advantage of the gratuitous, or almost gratuitous, services of hospitals and dispensaries; and this plan would be likely to increase their number largely.

The annual general meeting of the governors of the Cancer Hospital, Brompton, was held on the 22nd ult. G. S. Measom, Esq., J.P., occupied the chair, and amongst those present were: Captain Benyon, C. Burkitt, Esq., the Rev. G. Blunt, J. Carr, Esq., Sir Edward Hertolet, Dr. Marsden, E. S. Mounsey, Esq., Dr. Phené Harmer Steele, Sir Henry White, Sir F. B. Alston, Sir W. Farrer, G. S. Hinchliff, Esq., H. G. Eckford, Esq., P. Adlard, Esq., etc. The report, which was read by the secretary, stated that during the past year the number of new patients had been 1,715; 703 being admitted to the wards, and 1,012 treated as out-patients. The Samaritan Fund for assisting discharged patients had proved very useful, upwards of 200 patients having received help from this source. Some were sent to convalescent homes; others received pecuniary aid on leaving the hospital. The continued increase in the work of this institution calls for increased support, the reliable income being £3,000 a-year less than the ordinary expenditure. The terror which the very thought of cancer rouses in our breasts, should make all who can do so, help in relieving the sufferers from this terrible disease.

The following vacancies are announced this week, the amount of salary in each case being given after the name of the institution :—

**Resident Medical Officers.**

British Seamen's Hospital, Cronstadt, St. Petersburg. Resident Medical Officer.—£180 per annum.
Coton Hill Lunatic Hospital. Assistant Medical Officers.—£100 per annum, with board
East London Hospital for Children, Shadwell, E. Resident Clinical Assistant.—Board ; no salary.
Leicester Infirmary and Fever House. Assistant House Surgeon. £50, with board.
St. Helen's Friendly Societies' Medical Aid Association. Resident Medical Officer.

**Non-resident Medical Officers.**

Bristol Dispensary. Surgeon.
Central London Ophthalmic Hospital, Gray's Inn Road.
City of Aberdeen. Medical Officer of Health.—£300 per annum.
City of St. Albans. Medical Officer of Health and Analyst.—£65 per annum.
Clogher Union. Medical Officer, Aughnachy Dispensary.—£115 per annum, with fees.
Ludlow Union. Medical Officer, Munslow District.—£70 per annum, and fees.

**Nurses.**

Saughton Hall, Murrayfield, Midlothian. One, Trained.—£20.
Kingston Nurses' Home, Baker Street, Hull. One, Monthly, Surgical, and Medical.
Nursing Institution, Burton-on-Trent. One, Medical and Surgical.—£25.
Swansea and South Wales Nursing Institution. Several, Medical and Surgical.
Workhouse Infirmary Association, 6, Adam Street, Adelphi. Several.
Children's Hospital, Cardington, near Bedford. One.
Nursing Institution, Cheltenham. Several, Medical, Surgical, and Monthly.
Mr. D. E. Wilson's Institution, 96, Wimpole Street. Several thoroughly Trained Female and Male Nurses.
Memorial Hospital, Jarrow-on-Tyne. Two, with Surgical Training.
Bolton Infirmary and Dispensary. Two.
R. S. C. Hospital, Guildford. Two, Head Nurse and Assistant Nurse.
Nottingham and Notts Nursing Institution. One, Medical, Surgical, and Monthly.

**Probationer.**

Cottage Hospital, Batley, Yorkshire. One.

At the annual meeting of the subscribers to the Cumberland Infirmary, the Bishop of Carlisle and other speakers laid stress on the importance of getting the working men to contribute more largely to the funds of the hospital, and pointed to the good example of Workington, where the working men last year gave £600 to their new infirmary.

Mr. C. Giller, secretary of the People's Subscription Fund to the London Hospital, asks in a letter that East-end firms should consider the great importance of the hospital, and endeavour to maintain, if they cannot increase, their subscriptions, as the hospital is urgently in need of funds owing to the increase of patients, which last year numbered over 95,700.

Somewhat extensive structural additions are now in process of erection at James Murray's Royal Asylum, Perth. On both sides of the building new wards are being erected for the sick and infirm, and accommodation is also being provided for the more effectual individual treatment of recent and very acute cases. A small Turkish bath, and improved quarters for attendants, are also included in the new blocks. The elevation plans are pleasing and elegant, more ornate than the original buildings, but in harmony therewith. Ventilation is secured by extraction flues, the whole of the additions are to be heated by steam, and all internal details are of a most complete and finished description. It is expected that the new works will be completed by the end of the year, and as the new buildings are semi-detached the operations interfere but little with the amenity of the institution. The plans are by Mr. A. Heiton, F.R.I.B.A., Perth, under whom also the work is being executed. The estimates amount to about £7,000.

The Committee of the Essex and Colchester Hospital record in their annual report that 503 in-patients were admitted last year, of whom 112 were accident and emergency cases ; and the number of out-patients was 1,418. They also allude with regret to the deaths of two of their number, Messrs. James Inglis and Howard, the latter of whom was auditor to the hospital. Each of those gentlemen has bequeathed to it a legacy of £100.

In common with several of our contemporaries who have to be familiar with the position maintained by various journals from time to time, we were not surprised to read the announcement in Friday's *Globe* that the *Lancet* was about to change hands, or to see the statement contradicted the next evening. Matters are very different now from what they were when the editorial chair at the *Lancet* office was occupied by one of the ablest of medical journalists, as the *Medical Press and Circular* showed in a recent issue.

A charitably-disposed lady who appears to have been born on the first of days is desirous of endowing a Sunday cot at the Kilburn Hospital, for which purpose she solicits the donations of all who were ushered into the world on that day of the week. The ingenuity of the advertiser certainly deserves consideration, and seeing that there must be around us some hundreds of thousands of individuals who have a right to the privilege of giving under the conditions mentioned, she should have but little or no trouble in collecting all the money that is required for the purpose.

The report of the Newton Cottage Hospital and Dispensary shows a record of 79 in-patients and 566 out-patients treated during the year. Twenty of the in-patients came as accident cases of a more or less serious nature, and the medical report shows that some important operations took place, and many grave diseases were much benefited by the treatment given at the hospital. Of the five deaths that took place in the institution during last year, one occurred within ten minutes and one within twelve hours after admission ; so were clearly hopeless from the first. This leaves the number of deaths only three, a low rate of mortality, which must be satisfactory both to the nurses and to the medical officers. Two more gentlemen are to be added to the medical staff, so it may be expected that the hospital intends enlarging its sphere of operations.

The Leeds General Infirmary can still boast of an expenditure which does not exceed its income, but while the income was less last year than in 1886, the expenditure was more. One of the great mainstays of the infirmary is the amount contributed by the workpeople of the town, which came last year to £3,646 16*s.* 4*d.*, being £1,515 16*s.* 6*d.* more than in the previous year. Against this must be set the falling-off in the Hospital Sunday collections, from which the infirmary receives this year £1,799, a decrease of £302. As this decrease is coincident with a revival of trade in the town, it is a matter for more than regret. The annual report of the infirmary alludes with gratitude to the workpeople's Jubilee gift of two horse-ambulances, costing £328, already mentioned in our columns, and to the gift of £263 5*s.* 6*d.* presented as a Jubilee offering by the licensed victuallers of the town. The infirmary will soon be in possession of a most valuable branch institution in the shape of a convalescent home at Cookridge, the entire cost of which is being defrayed by Mr. John North. Mr. North's original donation for this purpose was £5,000 but learning that the building will cost more, he has generously intimated his intention of paying the remainder. This hospital will contain 42 beds, making 362 in all at the command of the infirmary, and will be ready for occupation in May. As last year the number of in-patients treated was 4,601, it may be guessed that the infirmary needs all the space it can secure ; but its prosperity and efficiency are a noble testimony to the generosity of the "north countrie."

